# Microarray-based ultra-high resolution discovery of genomic deletion mutations

**DOI:** 10.1186/1471-2164-15-224

**Published:** 2014-03-22

**Authors:** Eric J Belfield, Carly Brown, Xiangchao Gan, Caifu Jiang, Dilair Baban, Aziz Mithani, Richard Mott, Jiannis Ragoussis, Nicholas P Harberd

**Affiliations:** 1 Department of Plant Sciences, University of Oxford, South Parks Road, Oxford OX1 3RB, UK; 2 Wellcome Trust Centre for Human Genetics, University of Oxford, Roosevelt Drive, Oxford OX3 7BN, UK; 3 Max Planck Institute for Plant Breeding Research, Carl-von-Linné-Weg, Cologne 50829, Germany; 4 Department of Biology, LUMS School of Science and Engineering, Sector U-DHA, Lahore 54792, Pakistan; 5 McGill University and Genome Quebec Innovation Centre, 740 DR Penfield Ave, Montreal H3A 0G1, Canada

**Keywords:** Mutation, Deletion, Microarray, Genome, Comparative genomic hybridization, Probe density

## Abstract

**Background:**

Oligonucleotide microarray-based comparative genomic hybridization (CGH) offers an attractive possible route for the rapid and cost-effective genome-wide discovery of deletion mutations. CGH typically involves comparison of the hybridization intensities of genomic DNA samples with microarray chip representations of entire genomes, and has widespread potential application in experimental research and medical diagnostics. However, the power to detect small deletions is low.

**Results:**

Here we use a graduated series of *Arabidopsis thaliana* genomic deletion mutations (of sizes ranging from 4 bp to ~5 kb) to optimize CGH-based genomic deletion detection. We show that the power to detect smaller deletions (4, 28 and 104 bp) depends upon oligonucleotide density (essentially the number of genome-representative oligonucleotides on the microarray chip), and determine the oligonucleotide spacings necessary to guarantee detection of deletions of specified size.

**Conclusions:**

Our findings will enhance a wide range of research and clinical applications, and in particular will aid in the discovery of genomic deletions in the absence of *a priori* knowledge of their existence.

## Background

Oligonucleotide microarrays were first developed ~20 years ago [1]. Present day microarrays are vastly superior to their predecessors in terms of quality, probe density, and they can represent an entire species genome [2-4]. Microarray technology, together with advances in genomics and bioinformatics methodologies, revolutionized the ways that we interrogate and study genomes. These approaches have great power because they allow simultaneous survey and profiling of thousands of genes, and enable whole genomes to be assayed at particular moments in time and under specific conditions.

The scientific applications of microarray technology range from gene expression profiling, comparative genomic hybridization (CGH), and chromatin immunoprecipitation analysis, to single nucleotide polymorphism (SNP) detection. Recently, next generation sequencing (NGS) has also been used as a major discovery tool in these applications, particularly where well-annotated whole genome datasets are available [5]. However, for species with large, complex (e.g., transposon-rich [6]), polyploid genomes, or for species with genomes that are not well annotated, NGS is relatively poorly suited to the detection of SNPs, insertions/deletions (INDELs) and other variants because of the short sequencing reads and depth of sequencing coverage needed [7-9]. Moreover, the computational overheads associated with the analysis of NGS data are a significant barrier to their use in some laboratories. By contrast, microarrays are well-established research tools that require only well-established analysis methods. These can be performed easily on personal computers, and allow rapid and routine analysis of multiple samples. Hence, whilst the use of microarrays and NGS in genomic analysis will likely continue to be complementary [10], we here describe work specifically aimed at optimizing genomic deletion mutation detection via microarray-based approaches.

The first discovery of a plant genomic deletion mutation using microarray-based CGH utilized the *Arabidopsis thaliana* ATH1 genome array, a single-chip array featuring 22,500 probes representing approximately 24,000 *A. thaliana* gene sequences. CGH using this array led to the discovery of a phenotype-causal 523 bp fast-neutron (FN) irradiation-induced deletion mutation [11]. This deletion was within the second exon and intron of the *A. thaliana AtHKT1* gene (*At*4G10310) in a previously identified ion accumulation mutant [11]. Up to that point the identification of mutations that caused phenotypic changes in plants required laborious map-based cloning [12].

Mutagens such as FNs frequently induce small genomic DNA deletions of 1–6 bp [13] and permit the identification of phenotype-causal mutations in an unbiased manner versus mutagens such as EMS that almost exclusively cause G:C > A:T point mutations [12,14-17]. The use of tiling microarrays to discover these causal mutations potentially reduces the time, cost and labour needed, important when large numbers of samples are analysed [4,11]. However, mutation discovery remains a difficult process, especially with respect to relatively small-sized deletions.

In this study we describe the use of a graduated series of previously characterized *A. thaliana* genomic deletion mutations to discover the parameters required for rapid and robust detection of genomic deletion mutations, and in particular of relatively small sized deletion mutations. This series presents a range of deletion mutations, from 4 bp to ~5 kb, thus extending over a four orders of magnitude difference in deletion size. We use standard and customized versions of the Roche NimbleGen 2.1 million feature high-resolution microarrays [18] to detect these deletions via CGH, and are hence able to determine the oligonucleotide densities necessary to robustly detect genomic deletions as small as 4 bp in size. We show that this technology has the potential to uncover deletions that were not detectable with previous array based designs and equalling NGS-based technologies. Our findings may improve microarray design for a wide variety of applications [18].

## Results

### *Arabidopsis* oligonucleotide microarray design

**Figure 1:**
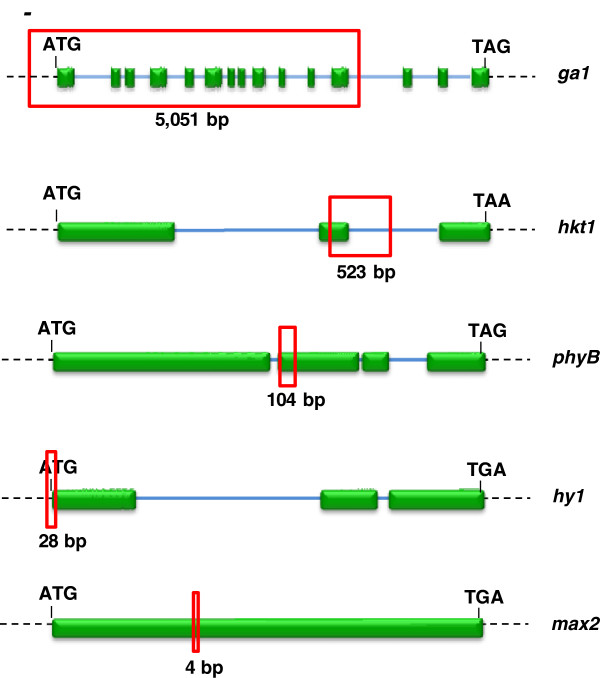
**A schematic showing the positions of five genomic deletions in five mutant plant lines.** The DNA deletions in mutants: *ga1-3*, FN1148, E124, E99 and E207 confer *ga1, hkt1, phyB, hy1* and *max2* mutant alleles, respectively. Green boxes represent exons, blue lines introns, dashed black lines inter coding sequences, and the start (ATG) and stop codons (TAG, TAA or TGA) for each gene are shown. The genomic deletion in each gene is shown as a red box with the size in bp indicated. The genes are not shown to scale.

The Roche NimbleGen custom *A. thaliana* CGH 3 × 720 K genome arrays can feature up to 2.16 million probes. The custom array that we designed featured the 2.09 million probes designed to the *Arabidopsis* TAIR8 annotated reference genome (http://www.arabidopsis.org) that represents 27,235 protein coding genes, 4,759 pseudogenes or transposable elements and 1,288 non-coding RNAs (33,282 genes in total and 38,963 gene models) at 49 bp staggering (the ‘standard’ array). In addition, ~15,000 ‘ultra-high density’ probes were added to the arrays to represent the genes *AtGA1, AtHKT1, AtPHYB, AtHY1* and *AtMAX2* and flanking genomic sequences (see Methods, Additional file 1: Tables S1 and S2). The single ‘ultra-high density’ probe set representing the larger deletions (> 500 bp in the mutant lines *ga1-3* and FN1148) were staggered every 6 bp apart (Additional file 1: Figure S1). Nineteen ‘ultra-high density’ probe sets were designed to represent the smaller deletions (≤ 104 bp) in the mutant lines E124, E99, and E207 and were staggered every: 2, 6, 10, 12, 15, 17, 20, 22, 25, 27, 30, 32, 35, 37, 40, 42, 45, 47 and 49 bp (Additional file 1: Figure S1). These additional features allowed us to determine the probe densities required for effective detection of the deletions of 4 bp to more than 5 kb present in the plant lines *ga1-3*, FN1148, E99, E124 and E207 (see Figure 1). The genomic deletions in E99, E124 and E207 were previously confirmed by direct PCR amplification (Additional file 1: Figure S2) and Sanger sequencing (Additional file 1: Table S3).

The arrays used in this study are unlike traditional tiling array formats that feature non-overlapping or partially overlapping probes with a maximum of one probe overlapping another. For example, in this study, the staggered probes designed every 2 bp can cover a single nucleotide position in the *Arabidopsis* genome up to 37 times, versus the maximum of twice using conventional tiling arrays. Probes containing sequences represented more than once in the genome were omitted from the final array design, and all probes in the ‘ultra-high density’ probe sets were unique within the TAIR8 annotated reference genome. The genomic distribution profiles of the ‘ultra-high density’ probes, like standard tiling arrays including the NimbleGen CGH arrays, are essentially linear and unbiased unlike most transcript arrays which are biased towards the 3′ end of transcripts and sometimes overlap [19]. This is because sequence data are typically derived from EST sequences with a 3′ bias, and the 3′ ends of genes are generally more variable and provide greater specificity [20].

### Microarray-based discovery of deletion mutations

Use of DNA (rather than mRNA) for microarray-based deletion cloning strategies has a number of benefits, as the mRNA abundance of many genes is too low to be efficiently and reliably detected. In addition, the relative levels of mRNA could be altered due to the secondary effects of the deletion mutation [11]. DNA from mutant plant lines or control lines labelled with Cy3 or Cy5 were co-hybridized to the CGH arrays. Genome-wide mutant DNA hybridization intensities were normalized with control genomic DNA samples hybridized to the same CGH array. Homotypic hybridizations (self-self: the same nucleic acid samples labelled with two fluorophores and hybridized to a single microarray) should result in a slope of Cy3 versus Cy5 intensity equal to one where normalized (Additional file 1: Figure S3). However, a number of putative deletions and/or copy number variants (CNVs) are always observed in such experiments as judged by a lack of hybridization intensity, or poor hybridization intensity of either the sample or control. Reduced hybridization signals are likely due to experiment-to-experiment variability and variation in the hybridization efficiency between individual probes [21,22].

**Figure 2:**
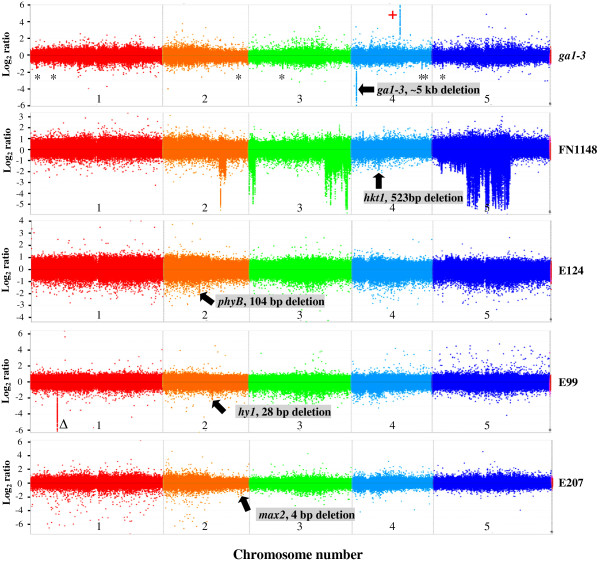
***Arabidopsis *CGH array data for mutant lines *ga1-3*, FN1148, E124, E99 and E207.** The CGH data is displayed as a single panel log_2_ signal ratio (mutant/control) rainbow plot for all probes plotted versus genome position, with each chromosome (1-5) differentiated by vertical dashed lines. Strongly negative points (e.g. −2 to −6) represent potential deletion mutations, strongly positive points (e.g. 2 to 6) represent potential duplication mutations. The known deletions in each mutant line are highlighted by an arrow and the name of the gene affected. Data is normalized and segmented. Putative deletions in *ga1-3* are shown with a ‘*’, and a confirmed deletion of ~7 kb in E99 is shown with a ‘∆’. The staggered ‘ultra-high density’ probes covering the larger deletions in the FN1148 (~500 bp *AtHKT1* genic mutation) and *ga1-3* (~5 kb *AtGA1* genic mutation) *Arabidopsis* mutants were staggered every 6 bp, while the extra probes representing the smaller deletions in the E124 (104 bp *AtPHYB* genic mutation)*,* E99 (28 bp *AtHY1* genic mutation) and E207 (4 bp *AtMAX2* genic mutation) *Arabidopsis* mutants were staggered every 2, 6, 10, 12, 15, 17, 20, 22, 25, 27, 30, 32, 35, 37, 40, 42, 45, 47 and 49 bp.

To detect the two largest deletions in our graduated mutant collection, *ga1-3* (~5 kb) and FN1148 (523 bp), we used a single probe staggering of 6 bp over each gene affected (in addition to the NimbleGen ‘standard’ probes staggered every 49 bp) (Additional file 1: Figure S1). Both deletions were observable at a genome-wide level (see Figure 2*ga1-3* and FN1148 profiles). The larger ~5 kb deletion in *ga1-3* at the *GA1* locus was visible as an obvious ‘deletion signal’ on chromosome 4 (Figure 2*ga1-3* profile). This deletion signal consisted of a set of neighbouring probes containing the *GA1* gene sequence that hybridized with control Ler DNA but failed to hybridize with *ga1-3* DNA, and matched exactly the previously established size and location of the *ga1-3* deletion [23]. The smaller deletion of 523 bp encompassing part of the *HKT1* gene [11] in the FN1148 mutant was also observable on a genome-wide scale on chromosome 4 (Figure 2 FN1148 profile).

Further analysis indicated the existence of seven other putative deletions displaying a probe ‘deletion profile’ threshold of 2 × S.D. in the *ga1-3* mutant genome (shown with a *, Figure 2; also see Additional file 1: Figure S4A). To determine if these were genuine deletions, we designed oligonucleotides to a gene within each deleted region and performed diagnostic PCR analyses. PCRs confirmed three of the seven putative deletions were false positives (on chromosomes 4 and 5) as the genes were amplified from both the Ler control and *ga1-3* mutant DNA samples, see Additional file 1: Figure S4B. We could not confirm the existence of the four deletions on chromosomes 1–3 as PCR products could not be amplified from either the control or mutant lines. Interestingly, we also observed a potential CNV (duplication) of ~4.5 kb on chromosome 4 (shown with a +; see Figure 2*ga1-3* profile), although it is alternatively possible that this signal represents a deletion event in the Ler control.

The most striking result of the FN1148 hybridization profile (versus control) was that there appeared to be a several large (~100 kb to 10 Mb) regions of chromosome 2, 3 and 5 that were deleted but no such deletions were observable on chromosomes 1 and 4 (Figure 2 FN1148 profile and Additional file 1: Figure S4C). Similar to the deletions identified in the *ga1-3* mutant versus control above, oligonucleotides were designed to six genes within these putative deleted regions and diagnostic PCR analyses performed to confirm authenticity. We did identify a deletion in the FN1148 mutant on chromosome 2 of ~4.5 kb (a PCR product of ~6 kb was amplified from the control plant DNA while the FN1148 mutant PCR product was ~1.5 kb), see Additional file 1: Figure S4D. This deletion spanned the entire 4 kb sequence of a transposable element (*At*2G31080). An alternative explanation for this deletion profile could be either the natural loss or movement of this mobile element in the FN1148 mutant genome. PCR analyses showed that the five other genes that appeared to be located in large deleted regions on chromosomes 2, 3 and 5 were actually present in both the control and FN1148 mutant lines, see Additional file 1: Figure S4D. These false positives could be associated with high signal-to-noise due to non-specific probe hybridizations or suboptimal technical aspects of the hybridization procedure such as sample labelling or preparation. Also, there is a possibility that there were DNA sequence specific differences between the *A. thaliana* FN1148 Col-0 mutant (obtained from J. Schroeder’s lab, UC San Diego, USA) and the control *A. thaliana* Col-0 line that had been propagated in our own laboratory since the 1990’s.

At a genome-wide scale, similar deletion profiles to the larger deletions (523 bp and ~5 kb) were observed for other *Arabidopsis* mutants with smaller deletions (4 bp, 28 bp and 104 bp). These deletion profiles were composed of nineteen staggered probe sets (2, 6, 10, 12, 15, 17, 20, 22, 25, 27, 30, 32, 35, 37, 40, 42, 45, 47 and 49 bp). Although the numbers of probes required to detect these smaller deletions was higher than those for the larger deletions, the normalized deletion signals visualized at the *PHYB, HY1* and *MAX2* loci (in E124, E99 and E207 mutant lines, respectively), were similar in magnitude to the 523 bp *hkt1* deletion (FN1148 mutant line) signal log_2_ ratio of ~ −2 but smaller than that observed for the *ga1-3* deletion (signal log_2_ ratio of ~ −6) (Figure 2). This was an interesting observation since the *PHYB, HY1* and *MAX2* loci DNA deletions varied in size by one to two orders of magnitude (the *MAX2* deletion mutation was 4 bp in size and the *PHYB* mutation was 104 bp), and as a consequence the number of probes representing these deletions was different. This suggests that there is not a simple relationship between deletion size and the ability to detect it. A total of 77 probes partially covered the *PHYB* deletion while the >25 times smaller deleted region of *MAX2* was represented 20 times in comparison. Also, the number of complete full-length probes covering these deletions differed: 23 covered the *PHYB* deletion but there were none representing the *MAX2* deletion, as the probe lengths were 50–75 mer and the size of the deletion just 4 bp.

Interestingly, we also detected an additional deletion mutation in the E99 mutant (versus control) on chromosome 1 using the standard NimbleGen probe set of 49 bp staggered probes (shown with a ∆; Figure 2). This deletion had a similar normalized signal log_2_ ratio of ~ −6 to the *ga1-3* deletion mutation. Oligos were designed to the flanking regions of this putative deletion and a PCR product was amplified (data not shown) from E99 genomic DNA confirming the deletion of 7,176 bp that encompassed three genes: *At*1G18075, *At*1G18080, and *At*1G18100, which encoded a microRNA, a DNA repair exonuclease and a protein of unknown function, respectively. In addition, the presence of this ~7 kb genomic deletion and a number of other mutations including the 28 bp deletion in the *HY1* gene, were confirmed by whole genome sequencing [13].

### In-depth analysis of probe densities required to detect relatively small genomic deletion mutations

To investigate the oligonucleotide probe resolutions required to detect genomic deletions smaller than those in *ga1-3* (~5 kb) and FN1148 (~0.5 kb) mutant lines, we compared the normalized hybridization profiles of individual probe datasets obtained for the E124 (104 bp *phyB* deletion), E99 (28 bp *hy1* deletion) and E207 (4 bp *max2* deletion) mutants. To do this we used nineteen probe sets staggered every 2, 6, 10, 12, 15, 17, 20, 22, 25, 27, 30, 32, 35, 37, 40, 42, 45, 47 and 49 bp (Additional file 1: Figure S1) over the affected genes, and determined the probe resolutions required to efficiently detect the different sizes of deletion.

**Figure 3:**
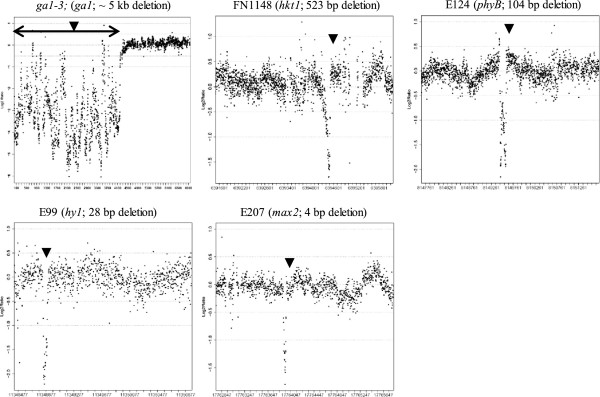
**Visualization of five genomic deletions of varying size in *Arabidopsis *mutant lines *ga1-3*, FN1148, E124, E99 and E207 (gene affected in each mutant and deletion sizes are shown).** Scatter plots show the relative log_2_ DNA hybridization intensities (mutant minus control) representing genes at 6 bp (*GA1* and *HKT1*) or 2 bp (*PHYB, HY1* and *MAX2*, see Additional file 1: Figure S5–8) probe spacing. Black dots represent probes and the black triangle indicates the location of the deletion within the region that the custom array probes represent. An obvious ‘deletion signal’ (contiguous points with relatively extreme negative log_2_ ratio values) was detected in all lines.

Scatter plots of the relative DNA hybridization intensity (mutant versus control), showed that the deletion mutations of 4 bp to 104 bp were observable as obvious deletion profiles using the lowest staggered probe staggering of 2 bp (Figure 3). Likewise, the single staggered probe set of 6 bp used to identify the deletions present in the *ga1-3* and FN1148 mutant lines were also observable as obvious deletion profiles (Figure 3). However, the cost of producing microarrays with oligonucleotides staggered every 2 bp (25-fold higher than that of the standard 49 bp NimbleGen CGH array) or 6 bp would be prohibitive, especially for species with large genomes.

**Figure 4:**
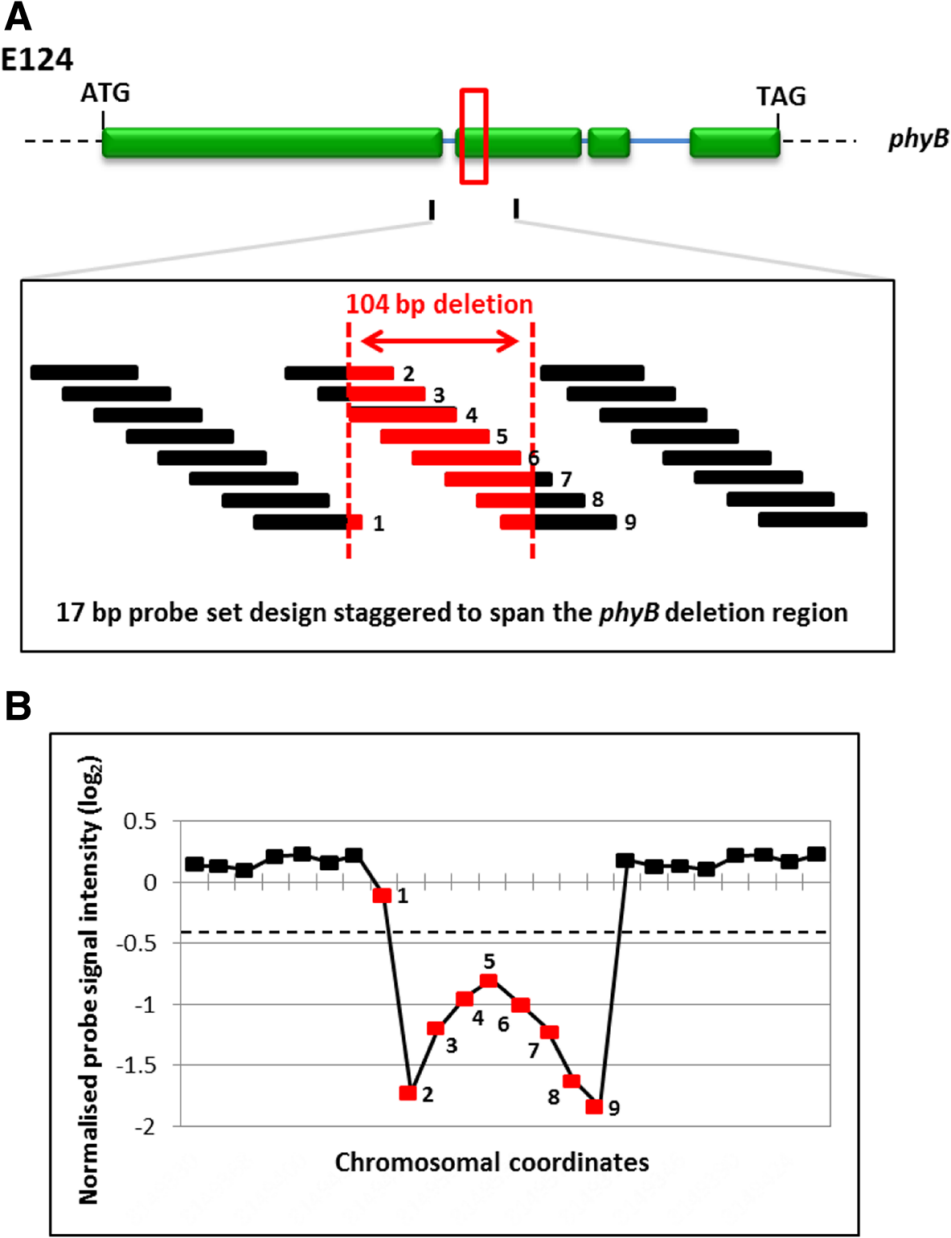
**The design and evaluation of probes used to detect a 104 bp genome deletion. (A)** A schematic showing the location of the 104 bp *phyB* deletion in the E124 *A. thaliana* mutant line. Also shown, an enlarged view of the 17 bp staggered probes designed to cover the deletion region. Nine probes cover the 104 bp deletion either fully (red filled boxes) or partially (black and red filled boxes). **(B)** A total of eight of the nine probes (89%; labelled 2 to 9) that covered the deletion region showed a typical deletion profile (normalized hybridization signal log_2_ ratio of less than −0.4 (dashed line)). One probe (number 1) had a log_2_ ratio value higher (−0.10506) than the −0.4 threshold.

**Figure 5:**
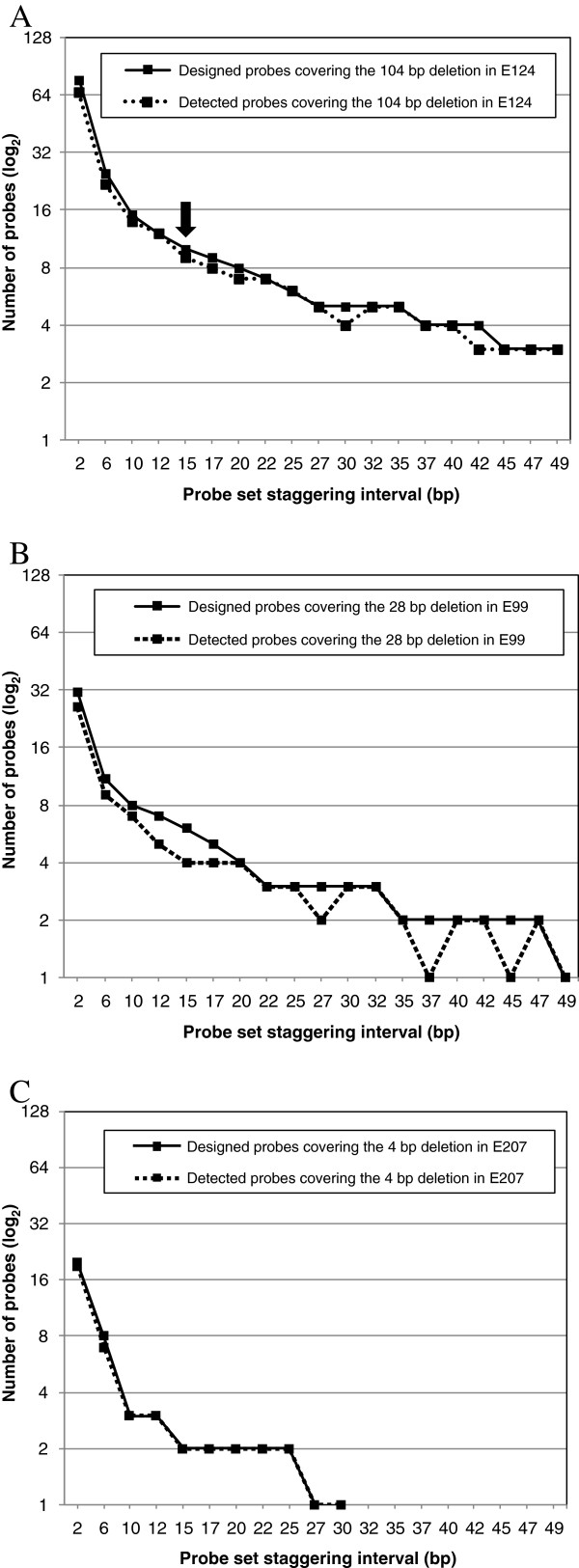
**The design and experimental performance of probe sets used to detect genome deletions. (A)** A graphical representation of the data shown in Table 1. The number of probes in each of the nineteen staggered probe sets ‘designed’ to cover the *phyB* deletion of 104 bp in E124 line are shown with a sold black line. The numbers of ‘designed’ probes, that were experimentally ‘detected’ with normalized log_2_ intensities of less than −0.4 are also shown with a dashed black line. For example, in Figure 4B 8 of the 9 (88.9%) designed probes (staggered every 17 bp) covering the *phyB* deletion had a normalized signal log_2_ ratio of less than −0.4 in Figure 4B. These 17 bp staggered probe numbers designed and detected are shown in **(A)** by a black arrow. **(B)** and **(C)**, are similar to **(A)**, the number of probes in each of the nineteen staggered probe sets designed to cover the *hy1* deletion of 28 bp in line E99 and the *max2* deletion of 4 bp in line E99 are shown with a sold black lines and those experimentally detected with normalized log_2_ intensities of less than −0.4 are also shown with a dashed black lines, respectively. The staggered probe data for the *max2* deletion (in line E207) >30 bp are not shown but the data is shown in Additional file 1: Figure S8.

In order to determine the density of probes required to detect smaller DNA deletions, we used a sliding window approach of two or three consecutive probes that had deletion profiles. To do this, the number of probes that either partially or fully covered the 4 bp to 104 bp deletions (termed ‘Designed’ probes), were compared to the number of probes following hybridization that had a deletion profile (termed ‘Detected’ probes). For example, of the 294 probes that were designed to span the whole *PHYB* gene with 17 bp staggered probes (Additional file 1: Table S2), only 9 either partially or completely covered the 104 bp deletion (in the E124 mutant line) (Figure 4A). Eight of these probes (88.9%), gave a detectable normalized deletion signal below the two S.D. threshold of ≤ −0.4, (see Methods, Figure 4B, Table 1 and Figure 5A).

**Table 1:** **The design and experimental performance of nineteen staggered probe sets used to detect a 104 bp genomic DNA deletion in an *A. thaliana *** **mutant**

**Probe set staggering (bp)**	**Number of probes**		**% of probes with a log**_**2 **_**normalized ratio of ≤ −0.4**
	**Designed**	**Detected with a log**_**2 **_**normalized ratio of ≤ −0.4**	
2	77	66	86
6	25	22	88
10	15	14	93
12	12	12	100
15	10	9	90
17	9	8	89
20	8	7	88
22	7	7	100
25	6	6	100
27	5	5	100
30	5	4	80
32	5	5	100
35	5	5	100
37	4	4	100
40	4	4	100
42	4	3	75
45	3	3	100
47	3	3	100
49	3	3	100

Table 1 The table shows the staggered design of probes that cover a 104 bp *phyB* deletion in the E124 *A. thaliana* mutant line. The number of probes in each staggered probe set that covered the deletion are shown (column titled ‘Designed’). The number and percent of ‘Designed’ probes that were experimentally detected with normalized log_2_ intensities of **≤ −**0.4 are also shown.

Further analysis was performed for all nineteen of the ‘Designed’ and ‘Detected’ ‘ultra-high density’ probe sets (2 bp to 49 bp) representing the *phyB* 104 bp deletion (in the E124 mutant line) and the results are listed in Table 1 and graphically shown in Figure 5A. Using a threshold of 3 consecutive probes that also had normalized log_2_ ratio intensities of ≤ −0.4 (i.e. a ‘deletion profile’), we found that probes staggered between 45 bp and the NimbleGen ‘standard’ probe density of 49 bp were sufficient to identify this size of deletion mutation (Figure 5A). We conclude that deletions of ~100 bp and larger can be confidently detected using tiling microarrays designed with isothermal probes staggered every 49 bp using a search criteria of ≥ 3 consecutive probes that have a deletion profile. Recently, a similar threshold of at least three consecutive probes with a normalized log_2_ ratio threshold of ≥ 0.4, has also been successful in identifying mutations (CNVs) in human patients with drug resistant epilepsy [24].

To detect smaller deletions of 28 bp (present in the E99 mutant line) and 4 bp (present in the E207 mutant line), higher probe densities than those used to detect the 104 bp above were needed. Figure 5B shows that probes would need to be staggered between 22 and 32 bp to detect the *hy1* 28 bp deletion, and between 10 and 12 bp to detect the 4 bp *max2* deletion (Figure 5C), using the same threshold of 3 consecutive probes with a deletion profile. However, if a less stringent threshold of just 2 consecutive probes was used, the 28 bp deletion was detectable using staggered probes of 35 to 47 bp, and likewise the 4 bp would be detectable using staggered probe resolutions of 15 to 25 bp.

Previous studies have shown that one or two probes are sufficient to detect genomic deletions of ~100 bp [4] and ~500 bp [11], deletions that are considerably larger than the smallest deletion (4 bp) analyzed in our study. Our analyses of the ‘standard’ probes on the NimbleGen arrays showed they were highly sensitive and highly reliable in detecting deletions of various sizes. For example, the 104 bp *phyB* deletion (E124 mutant) was represented on our custom microarrays by three probes at the ‘standard’ staggering of 49 bp (Table 1, Figure 5A and Additional file 1: Figure S5), while the smaller 28 bp and 4 bp deletions in the *hy1* (E99) and *max2* (E207) mutants were each represented just once at the same ‘standard’ staggering, respectively. However, all five of these ‘standard’ probes that represented these three different sized deletions gave normalized DNA hybridization log_2_ ratio values of less than −0.4 (Figures 5B and C, and Additional file 1: Figures S6 and S7). This shows the excellent sensitivity of this type of microarray, but the detection of a genomic deletion with just a single probe risks background signal-to-noise interference, with the persistent possibility of false positives being called.

Overall, the ‘ultra-high density’ probe sets used in our study enabled us to determine the number of probes required and at the precise staggering (between 2 and 49 bp) that allow reliable detection of deletions of just a few bp in size. These results will aid researchers designing microarrays to detect deletions of different sizes without any *a priori* knowledge of the deletion. For example, using a stringent 3 consecutive probe criteria, deletions of ~100 bp require microarrays with probes staggered ≤ 45 bp while smaller deletions of ~30 bp need probes staggered ≤ 32 bp and deletions of a just a few bp require probes staggered ≤ 12 bp (Figures 5A–C).

### NimbleGen microarray performance analysis

**Figure 6:**
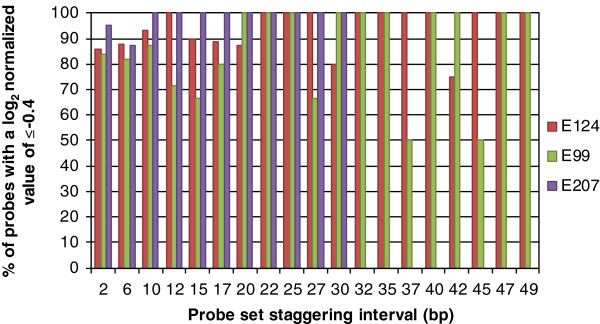
**A histogram showing the percentage of ‘designed’ probes that indicated a genomic deletion.** The columns represent the percent of total probes that covered each of the deleted regions in lines E124 (104 bp *AtPHYB* genic mutation)*,* E99 (28 bp *AtHY1* genic mutation) and E207 (4 bp *AtMAX2* genic mutation) that were detected as deletions. The probes that were categorized as deletion probes had normalized hybridization signal log_2_ ratios (mutant versus control) of ≤ −0.4.

A previous study used an additional criterion to predict deletions in rice plants using microarrays based on a combination of probe threshold and probe proportions [25]. Bruce *et al*., (2009) called putative deletions when ≥ 50% of the probes representing a gene model had probe log_2_ ratio intensities (mutant probe intensity/wild type probe intensity) of ≤ −0.8. In our study, all nineteen ‘ultra-high density’ probe sets (2, 6, 10, 12, 15, 17, 20, 22, 25, 27, 30, 32, 35, 37, 40, 42, 45, 47 and 49 bp) used to detect the 4 bp, 28 bp and 104 bp deletions had ≥ 50% of probes with log_2_ ratio intensity ratios below the two S.D. threshold of ≤ −0.4, indicative of genomic deletions (Figure 5A–C, and Figure 6). We found the average number of ‘detected’ probes with a ‘deletion profile’ out of all nineteen ‘designed’ ‘ultra-high density’ probe sets were: 94%, E124; 86%, E99; and 99%, E207 (Figure 6). For example, 95% (19 of 20 probes) of the 2 bp staggered probes representing the smallest deletion (4 bp present in the E207 mutant line) had a log_2_ ratio normalized intensity of less than −0.4 (Figure 6). Likewise, 84% (26 of 31) and 86% (66 of 77) of the 2 bp staggered probes covering the two larger deletions (28 bp and 104 bp present in the E99 and E207 mutant lines, respectively) had a log_2_ ratio normalized intensity of less than −0.4 (Figure 6). Although, the lowest values we observed were 50% for two out of the fifty-seven ‘ultra-high density’ probe sets (the 37 bp and 45 bp probe sets representing the *hy1* mutation in E99; Figure 6), the overall results suggest that most of the ‘ultra-high density’ NimbleGen CGH probe sets used in our study are highly sensitive, and are efficient in detecting deletion of various sizes.

## Discussion

Microarrays are a powerful tool in the genetic analyses of both prokaryotes and eukaryotes. In this study we determined the probe resolution required on microarrays to reliably detect genome deletions. Using irradiated *A. thaliana* plant lines that were isolated from forward genetic screens, we were able to identify mutant phenotype-causal deletions that ranged in size from just a few bp to larger kb variants. Although forward genetics has been used for almost 100 years, a major difficulty of this technique is the molecular identification of the causal mutations responsible for an observed phenotype. This is especially true for smaller deletions of just a few nucleotides that can remain undetected.

In order to study the appropriate density of probes on a microarray to detect small genomic deletions, we utilized a set of *A. thaliana* mutant plant lines that we had previously characterized. These mutants, obtained from an irradiation mutagenesis screen, had genomic deletions in three photomorphogenesis genes *MAX2*, *HY1*, and *PHYB*. The deletions were 4 bp to 104 bp in size and made excellent test cases to optimize CGH-microarray based genomic deletion detection. We designed a NimbleGen deletion profiling microarray to detect these *A. thaliana* mutations. This targeted array contained ultra-high density probes covering the entire genomic regions of the three genes affected and included exon, intron, promoter and UTR sequences. The probes were added to the microarray in nineteen physically staggered designs to cover each region from 2 bp to 49 bp. Following normalization of the hybridization signals of the mutants versus control samples, we could perform in-depth analysis of the probe densities required to confidently detect relatively small genomic deletions.

We found that to detect genomic deletions of ~100 bp, a microarray with probes staggered every 45 bp or less was required (Figure 5A). We also found that smaller deletions of 28 bp and 4 bp required higher densities of probes of at least 22 bp and 10 bp, respectively (Figure 5B and C). It is important to note that the long oligonucleotide probes (60–80 mer) on the NimbleGen microarrays we used in this study provided excellent detection sensitivity and reliability (see Table 1, Figures 5A–C and Figure 6). Microarrays from alternative manufacturers with shorter probes (e.g. 25 or 30 mer) might not have detected similar deletions because they have been shown to be less sensitive than longer probes [26,27].

Although most microarray applications are research-use-only, this technology is increasingly being used in clinical based genomic applications [28]. For example, microarrays have been used to identify DNA deletions associated with serious human conditions such as cancer [29], muscular dystrophy [30], and osteoporosis [31]. In addition, personalized healthcare is a rapidly developing scientific area and microarrays may be utilised in the identification and early detection of treatable diseases [28]. The deletions reported in these previous clinical studies vary in size from 100's of bases to 100's of kilobases. While these studies demonstrate that arrays are an excellent tool for identifying DNA deletions, the density of oligonucleotides required to detect smaller disease-causing deletions remains unclear.

With the advent of NGS it is now possible for genomes to be sequenced at high depth. However, at the present time no single platform, neither NGS nor microarrays, can identify all genomic sequence variants [32]. NGS can be very expensive, computational costs can be high and it can be time prohibitive for large numbers of samples [33]. Indeed, whole-genome sequencing is not necessary for many research studies that focus on specific target regions, such as promoters, exons and regulatory elements. This is also true for genomes with highly repetitive DNA sequences, such as the human and wheat genomes that are composed of 50% and 80% repeats, respectively [34,35]. For example, the 50 Mbs of the extended human exome could easily be represented on a single array [36]. Microarrays are a less labour intensive alternative to NGS and are likely to remain popular in research laboratories especially for those without extensive bioinformatic support. Also, microarrays can provide rapid and parallel analysis of large numbers of samples. Oxford Gene Technology, for example, processed 20,000 samples in 20 weeks on behalf of The Wellcome Trust Case Control Consortium researching CNVs in common human diseases [32].

## Conclusion

The small deletions analysed in this report are typical of those found in plants following irradiation mutagenesis [13] and in certain human disorders: frameshift causing deletions of 4 bp have been reported to be responsible for severe brain malformations [37]; a condition in families that can cause the lung to collapse [38]; and in a syndrome that can lead to increased risk of cancer of the colon, stomach, and pancreas [39]. Our findings show that the probe density on a microarray is critical in identifying genomic deletions and is fundamental to the success of experiments. Our results will help researchers working on both prokaryotes and eukaryotes to design microarrays with the optimal probe densities to detect both large and small deletions. These findings are applicable to any organism with a well-annotated sequenced reference genome.

## Methods

### Plant material and growth conditions

All experiments used either the Landsberg *erecta* (Ler) or the Columbia (Col-0) laboratory strains of *A. thaliana* as genetic background. *ga1-3* seeds were originally isolated from a FN-mutagenized Ler plant [40,41] that had a genomic deletion encompassing part of the *GA1* gibberellin biosynthesis gene (*At*4G02780). Seeds of the Col-0 *AtHKT1* mutant (FN1148) were kindly donated by Julian Schroeder, UC San Diego, USA [11]. Plants were grown on soil with a 16 h light/8 h dark photoperiod at 22–24°C (irradiance 120 μmol photons m^−2^ s^−1^).

Three *Arabidopsis* elongated hypocotyl mutants E99, E124 and E207 were obtained from visual screens of seedlings grown from a fast neutron irradiated DELLA deficient (*gai-t6, rga-t2, rgl1-1, rgl2-1* and *rgl3-4*) [42] seed collection [13]. The plants harbour genomic deletions within the *HY1* (*At*2G26670) [40], *PHYB* (*At*2G18790) [43] and *MAX2* (*At*2G42620) [44] genes, respectively. The genomic deletion in each FN elongated hypocotyl mutant was confirmed by direct PCR amplification and Sanger sequencing using the oligonucleotides listed in Additional file 1: Table S3. Table S1 (see Additional file 1) gives an overview of the five *Arabidopsis* mutants used in this study, the sizes of the fast neutron-induced deletions, the coordinates of the deletions, and the gene affected.

### DNA extraction and microarray experiment

Genomic DNA was extracted from plant leaf material using a DNeasy Mini Kit (Qiagen). The yield and quality of the samples was checked by running the samples on a 1% agarose gel (data not shown), and 6 μg was sent to Roche NimbleGen’s custom microarray services facility. Microarray hybridizations and washings were performed using the standard NimbleGen protocol for CGH analyses (http://www.nimblegen.com/products/cgh/index.html).

Control DNA samples were extracted from *A. thaliana* Col-0 and used to normalize the FN1148 mutant hybridization signal; wild type *A. thaliana* Ler DNA was used to normalize the *ga1-3* mutant hybridization signal; and the DNA of the DELLA-deficient progenitor line (mostly *A. thaliana* Ler background with a ~3 Mb chromosome 5 Col-0 segment [13] was used as the control to normalize the E99, E124 and E207 mutant hybridization signals.

### Microarrays

We used Roche NimbleGen’s two-colour *Arabidopsis* CGH 3 × 720 K whole genome custom tiling arrays that feature empirically tested probes of 50–75 mer that provide improved data quality (i.e. signal-to-noise) compared with computationally selected probes (http://www.nimblegen.com/products/cgh). Previous studies have shown that microarrays with longer oligonucleotides (60–80 mer) provide significantly better detection sensitivity than those with shorter oligonucleotides (e.g. 25 or 30 mer) [26,27,45].

Using the *A. thaliana* TAIR8 annotated reference genome sequence (http://www.arabidopsis.org), probes were designed to represent the complete model plant genome every 49 bp with 50–75 bp partially overlapping probes, i.e. the distance between the 5′ ends of consecutive oligonucleotides. In addition to these ‘standard’ array probes staggered every 49 bp, extra probes were added with the purpose of representing the five genes *AtGA1, AtHKT1, AtPHYB, AtHY1* and *AtMAX2* at ‘ultra-high density’. To achieve this purpose, around 15,000 isothermal ‘ultra-high density’ probes (50–75 bp; see Additional file 1: Table S2) were designed to cover the genomic regions of each of the five genes from 500 bp 5′ to the gene’s start codon, continuing through the genic region (including both exonic and intronic sequences) and ending 500 bp 3’ of the stop codon.

Probes were designed to represent the larger deletions in the FN1148 (~500 bp *AtHKT1* genic mutation) and *ga1-3* (~5 kb *AtGA1* genic mutation) *Arabidopsis* mutants with oligos staggered every 6 bp, while the extra probes representing the smaller deletions in the E124 (104 bp *AtPHYB* genic mutation)*,* E99 (28 bp *AtHY1* genic mutation) and E207 (4 bp *AtMAX2* genic mutation) *Arabidopsis* mutants were staggered every: 2, 6, 10, 12, 15, 17, 20, 22, 25, 27, 30, 32, 35, 37, 40, 42, 45, 47 and 49 bp (Additional file 1: Figure S1; Additional file 2: Tables S4–S22). To reduce the background noise of the ‘ultra-high density’ probes, probes that mapped perfectly to more than one genomic location were excluded. Also, sequences that were unique to the *Arabidopsis* TAIR8 annotated reference genome were assigned randomized locations across the NimbleGen array.

### Microarray hybridizations and data analysis

Test and reference control genomic DNAs were independently labelled with fluorescent dyes (mutant DNA was labelled with cyanine (Cy) 3 and control DNA labelled with Cy5), co-hybridized to the NimbleGen *A. thaliana* 2.1 M Whole-Genome CGH arrays, and scanned using a 5 μm scanner. Log_2_-ratios of the probe signal intensities (Cy3/Cy5) were calculated and plotted versus genomic position using Roche NimbleGen NimbleScan software. To determine the efficiency and to reduce costs associated with CGH-based deletion discovery, we used a single array per deletion experiment without replicate hybridizations.

The criteria used to identify deletions and CNVs by aCGH vary considerably between studies. Such mutations are commonly distinguished from low-level gains/losses using a direct threshold of array data. However, the threshold value often differs greatly, ranging from a log_2_ ratio of +/−0.4 for some studies [46,47] to as high as +/−1.0 for others [48,49]. The criteria we used to detect deletions were based on the aCGH patterns obtained from our mutant versus wild type hybridizations. From our E124, E99 and E207 versus control hybridizations, probes that represented loci with an equal copy number, had a mean log_2_ normalized intensity ratios of 0.0 +/−0.2 S.D. Based on this variation, empirical analyses (based on looking at the frequency distribution of log ratios for probes along known deleted and duplicated regions), and previous studies [50,51], we chose a probe ‘deletion profile’ threshold of 2 × S.D., i.e. +/−0.4. Indeed, of the total number probes on the microarrays only 2.73 – 4.97% from each mutant versus control hybridization gave log_2_ ratios above or below +/−0.4, suggesting the probes on the microarray had a low level of signal-to-noise. In addition, we would expect about 5% of probes to exceed this threshold by chance if the log2 ratios are normally distributed.

### Availability of supporting data

The data sets supporting the results of this article are available in the NCBI GEO repository (accession number GSE55327 study at: http://www.ncbi.nlm.nih.gov/geo/query/acc.cgi?acc=GSE55327).

## Abbreviations

CGH: Comparative genomic hybridization; CNV: Copy number variation; Cy: Cyanine; FN: Fast-neutron; INDELs: Insertions/deletions; NGS: Next generation sequencing; SNP: Single nucleotide polymorphism.

## Competing interests

The authors declare that they have no competing interests.

## Authors’ contributions

EJB, RM, JR and NPH conceived the experiment. EJB, CB and CJ grew plants and extracted genomic DNA. DB provided microarray technical expertise and interpretation of data. AM and XG provided bioinformatics support. EJB and NPH wrote the manuscript. All authors read and approved the final manuscript.

## Supplementary Material

Additional file 1: Figure S1 A schematic showing the staggered probe sets used to detect genomic deletions of various sizes. **Figure S2.** PCR analyses of genomic deletions present in FN mutant plant lines E124, E99 and E207. **Figure S3.** The distribution of the ‘ultra-high density’ probe sets over five genomic regions represented on the Roche NimbleGen *A. thaliana* CGH array. **Figure S4.** PCR analyses of putative genomic deletions identified in mutant lines *ga1-3* and FN1148. **Figure S5.** A 108 bp deletion located in the *phyB* gene of the E124 mutant detected with NimbleGen CGH arrays. **Figure S6.** A 28 bp deletion located in the *hy1* gene of the E99 mutant detected with NimbleGen CGH arrays. **Figure S7.** A 4 bp deletion located in the *max2* gene of the E207 mutant detected with NimbleGen CGH arrays. **Figure S8.** Design and experimental performance of the NimbleGen CGH array probes. **Table S1.** A table showing the details of known deletions in five *Arabidopsis* mutants used in this study. **Table S2.** The table lists the numbers of custom array probes staggered from 2 bp to 49 bp representing the genes in five *Arabidopsis* deletion mutants used in this study. **Table S3.** List of genomic DNA deletion mutations identified and verified in three fast-neutron irradiated mutants. Click here for file

Additional file 2: Tables S4–S22 List and design information for custom probes with 2 to 49 bp staggerings. Click here for file
