# Supplementary material for: Microarray-based ultra-high resolution discovery of genomic deletion mutations
Source: BMC Genomics. 2014 Mar 22;15:224. doi: 10.1186/1471-2164-15-224 (PMC3998191; doi:10.1186/1471-2164-15-224)
Supplement: Additional file 1: Figure S1 — A schematic showing the staggered probe sets used to detect genomic deletions of various sizes. Figure S2. PCR analyses of genomic deletions present in FN mutant plant lines E124, E99 and E207. Figure S3. The distribution of the ‘ultra-high density’ probe sets over five genomic regions represented on the Roche NimbleGen A. thaliana CGH array. Figure S4. PCR analyses of putative genomic deletions identified in mutant lines ga1-3 and FN1148. Figure S5. A 108 bp deletion located in the phyB gene of the E124 mutant detected with NimbleGen CGH arrays. Figure S6. A 28 bp deletion located in the hy1 gene of the E99 mutant detected with NimbleGen CGH arrays. Figure S7. A 4 bp deletion located in the max2 gene of the E207 mutant detected with NimbleGen CGH arrays. Figure S8. Design and experimental performance of the NimbleGen CGH array probes. Table S1. A table showing the details of known deletions in five Arabidopsis mutants used in this study. Table S2. The table lists the numbers of custom array probes staggered from 2 bp to 49 bp representing the genes in five Arabidopsis deletion mutants used in this study. Table S3. List of genomic DNA deletion mutations identified and verified in three fast-neutron irradiated mutants. [file 1471-2164-15-224-S1.docx]

**Additional file 1**

**Figure S1**


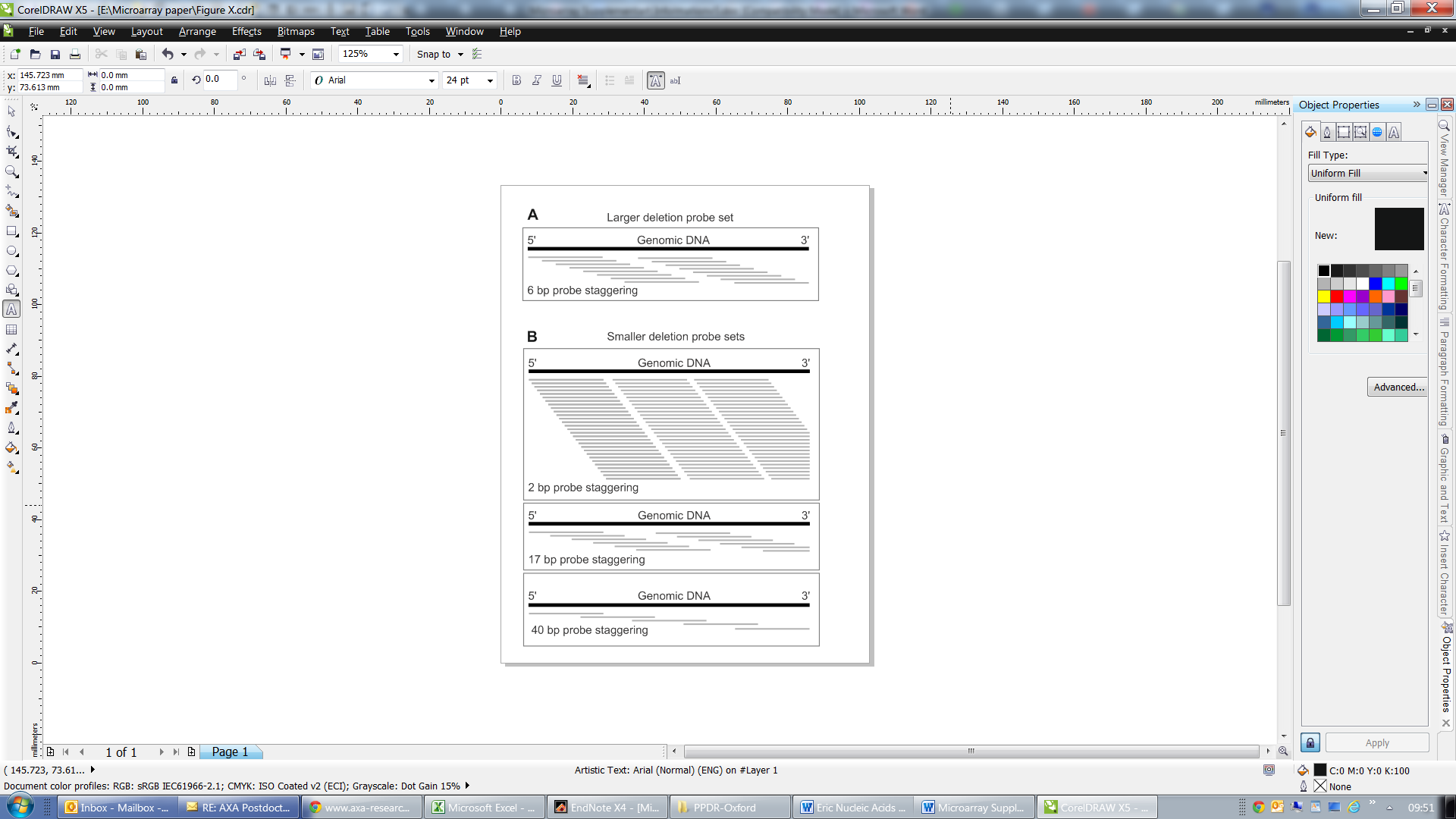


**Figure S1**. A schematic showing the staggered probe sets used to detect genomic deletions of various sizes. (A) To detect larger deletions of ~5 kb (present in the *ga1* gene of mutant line *ga1-3*) and 523 bp (present in the *hkt1* gene of mutant line FN1148) we used a single staggered 6 bp probe set to represent each gene. The staggered probes are shown as grey boxes and the genomic region of DNA the probes represent is shown as a black box (B) To determine the density of probes required to efficiently detect smaller deletions of 104 bp (present in the *phyB* E124 mutant line), 28 bp (present in the *hy1* E99 mutant line) to 4 bp (present in the *max2* E207 mutant line), nineteen staggered probe sets were designed every 2, 6, 10, 12, 15, 17, 20, 22, 25, 27, 30, 32, 35, 37, 40, 42, 45, 47 and 49 bp over the gene affected. Three of the nineteen staggered probe sets (2 bp, 17 bp, and 40 bp) are shown in (B).

**Figure S2**

**A B**

**
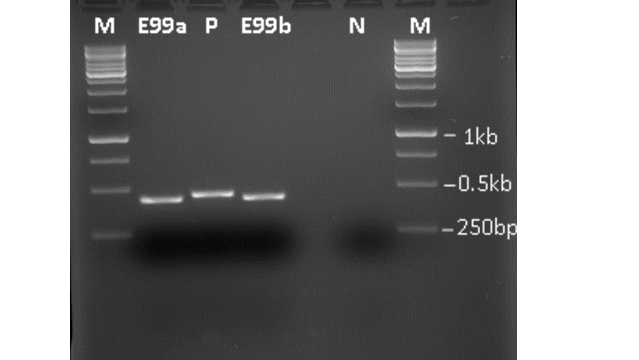

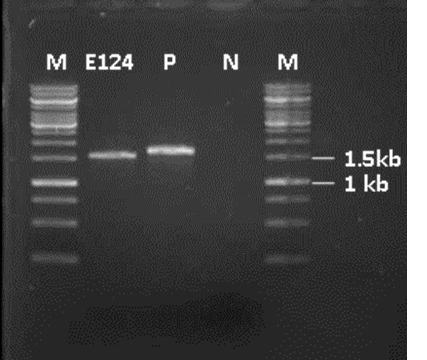
**

**C**


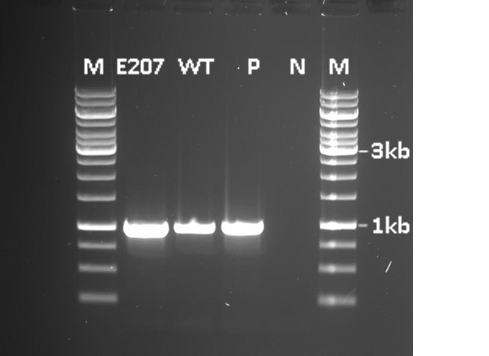


**Figure S2.** PCR analyses of genomic deletions present in FN mutant plant lines E124, E99 and E207. Photos of agarose gels showing smaller sizes of PCR amplified products can clearly be observed in (A) E124 (*PHYB* gene deletion of 104 bp) and (B) duplicate lines E99a/b (*HY1* gene deletion of 28 bp) versus control (progenitor line (P)) samples. The 4 bp deletion in E207 (*MAX2* gene deletion) could not be resolved using gel electrophoresis (C) versus control (P) or wild type (WT, *A. thaliana* Ler) samples due to the small deletion size. N = negative PCR control and M = DNA size marker. All deletions were confirmed by Sanger sequencing of the PCR products shown.

**Figure S3**

**
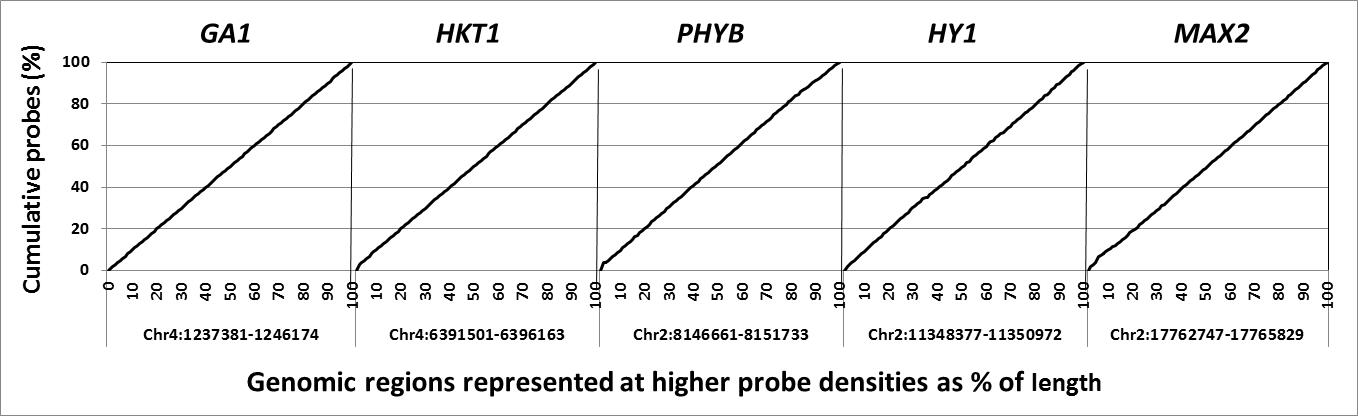
**

**Figure S3**. The distribution of the ‘ultra-high density’ probe sets over five genomic regions represented on the Roche NimbleGen *A. thaliana* CGH array. The genomic regions (Chromosome and coordinates): Chr4:1237381-1246174, Chr4:6391501-6396163 Chr2:8146661-8151733, Chr2:11348377-11350972, and Chr2:17762747-17765829, represent the *GA1, HKT1, PHYB, HY1,* and *MAX2* genes, respectively. The lengths of the genomic regions are not equal because the absolute lengths of the gene represented in each dataset are different (for example *GA1* is 6885 bp in size while *HY1* is 1578 bp). The regions are shown as a percentage of length.

**Figure S4**

**A**


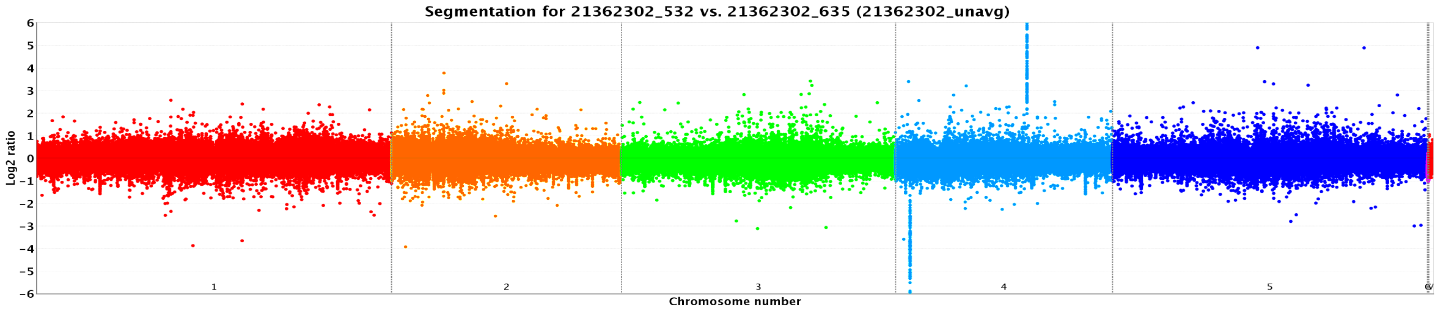


2

4

-

2

0

-

6

-

4

6

***ga***

***1***

***-***

***3***

***ga1***

***-***

***3***

**+**

1

2

4

3

5

**Log_2_ ratio**

**Chromosome number**

Del 1

Del 2

Del 3

Del 4

Del 5

Del 6

Del 7

**B**


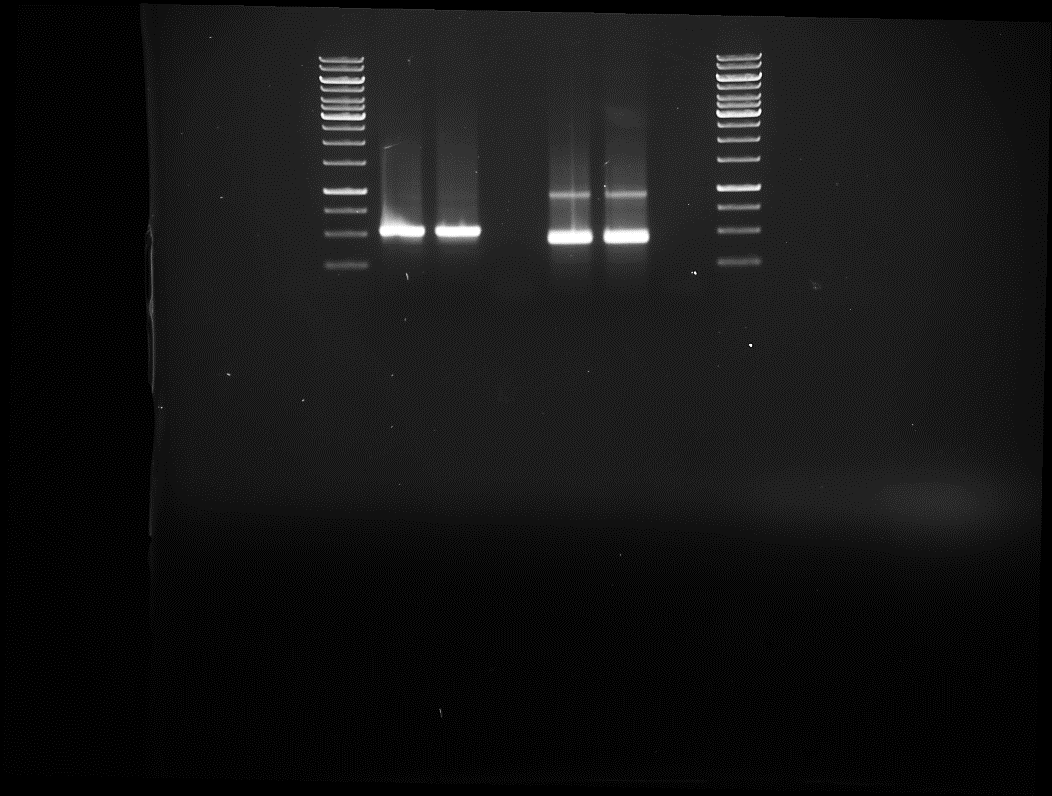

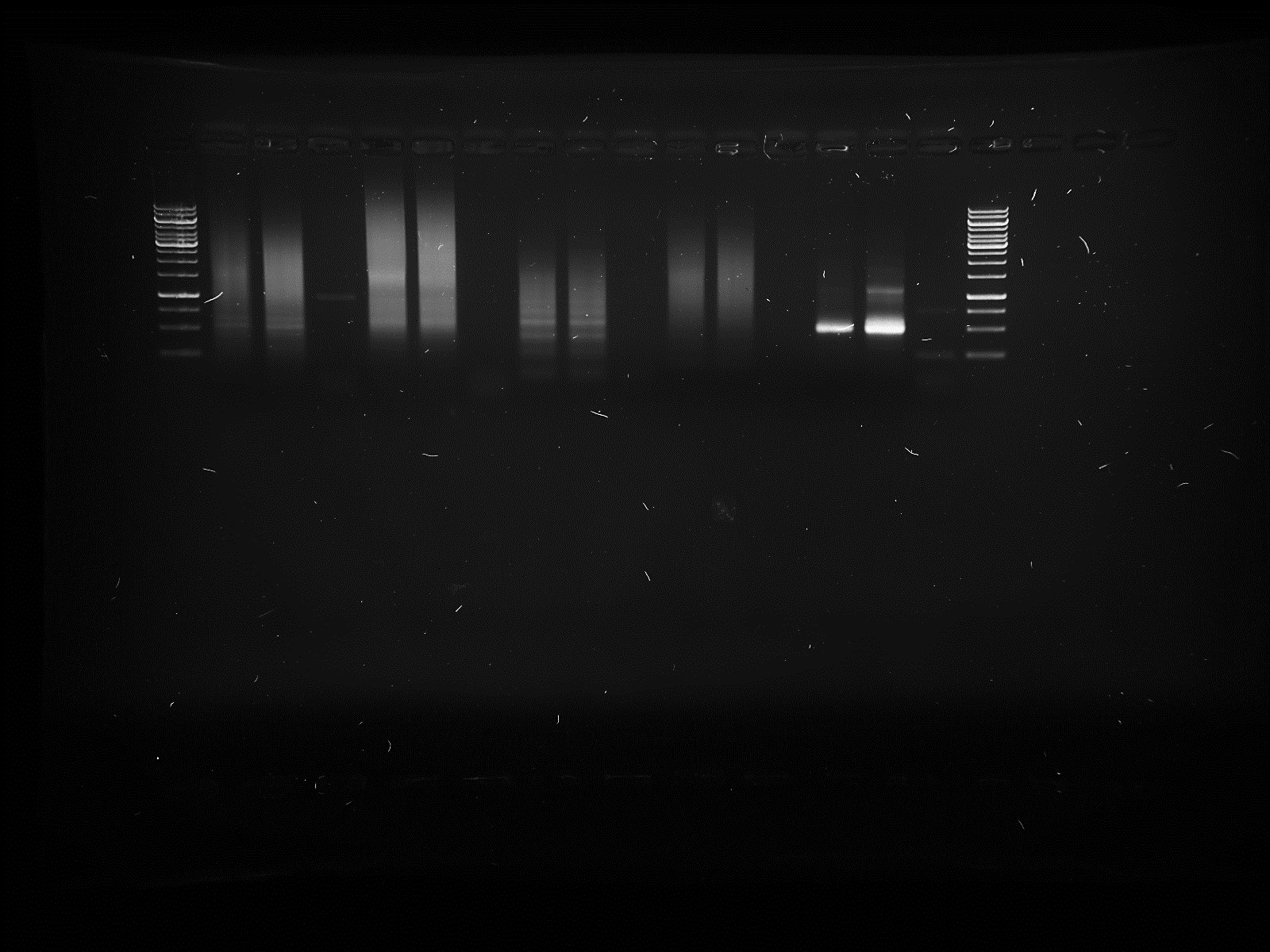


Del 5

WT Mut N

Del 6

WT Mut N

Del 7

WT Mut N

2 kb

0.5 kb

1 kb

M

M

M

**C**

**Log_2_ ratio**


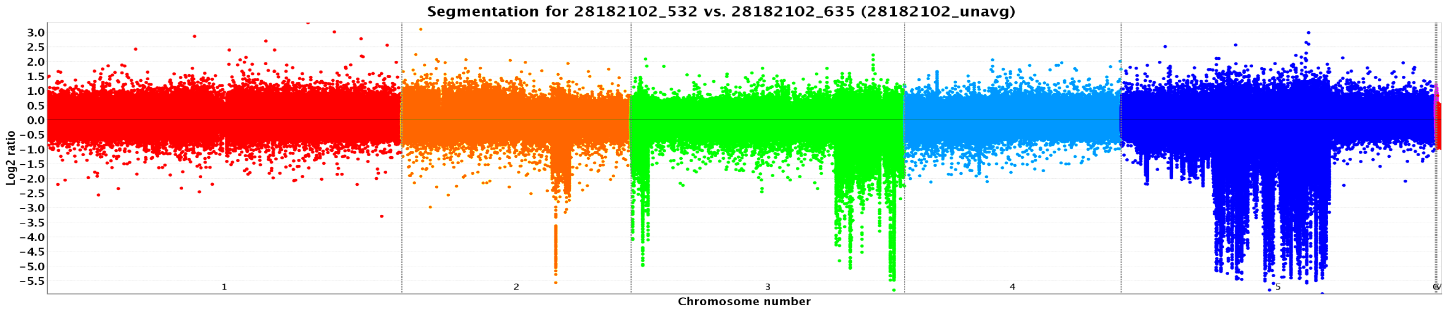


2

3

-

2

0

-

4

-

5

1

-

3

-

1

**FN1148**

1

2

4

3

5

**Chromosome number**

Del 1

***hkt1***

Del 2

Del 3

Del 4

Del 5

Del 6

**D**


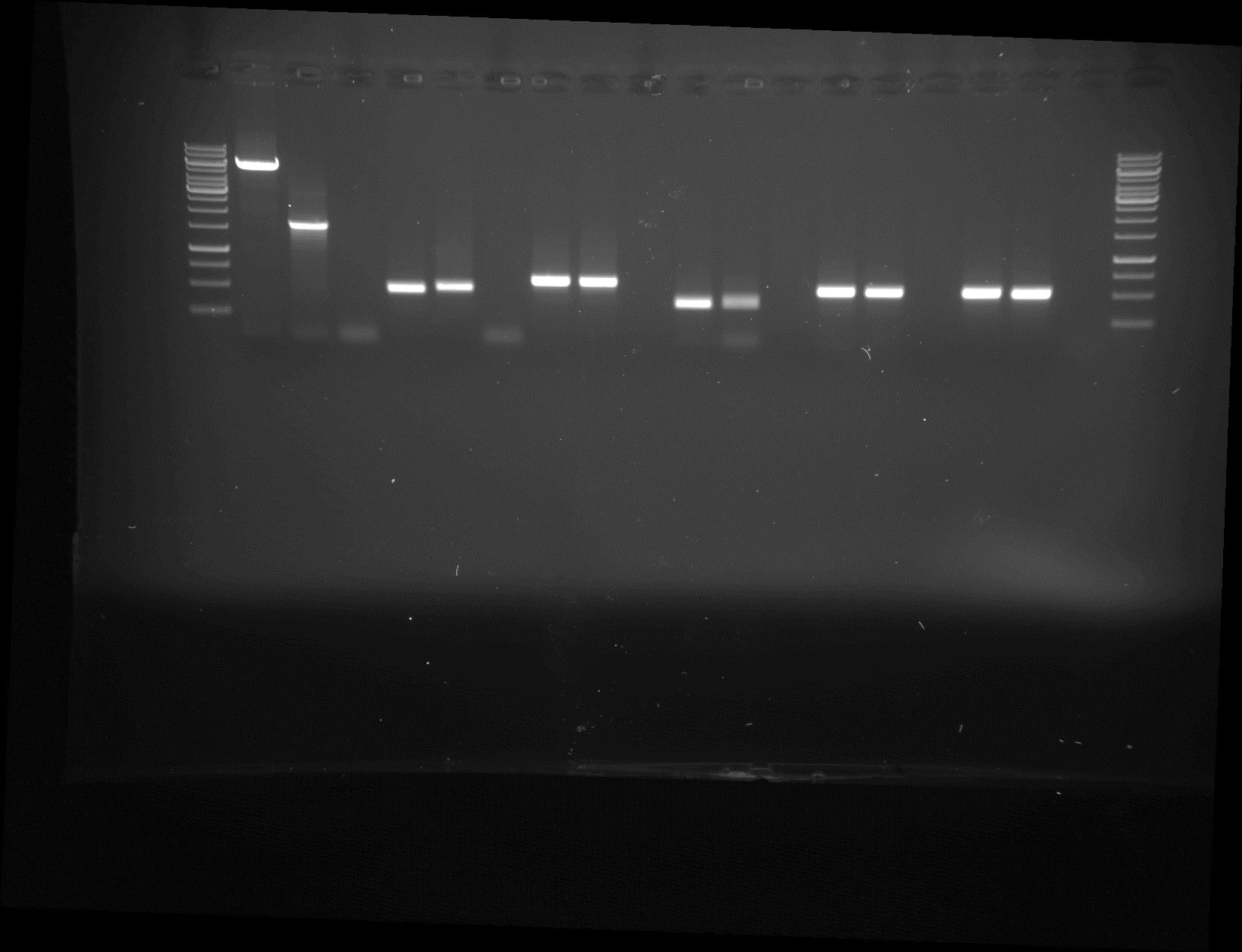


6 kb

2 kb

0.5 kb

1 kb

M

M

Del 1

WT Mut N

Del 2

WT Mut N

Del 3

WT Mut N

Del 4

WT Mut N

Del 5

WT Mut N

Del 6

WT Mut N

**Figure S4.** PCR analyses of putative genomic deletions identified in mutant lines *ga1-3* and FN1148. (A) Shows the *ga1-3* hybridization profile versus control CGH data (displayed as a rainbow plot panel log_2_ signal ratio (mutant/control)), from Figure 2. Initial observations suggested the presence of seven genomic deletions (Del 1–7) on chromosomes 1–5 in the *ga1-3* profile (these probes displayed a ‘deletion profile’ threshold of 2 x S.D.). Oligonucleotides were designed to genes within these deleted regions and diagnostic PCR analyses performed. (B) An example agarose gel photograph of the PCR products amplified from the *ga1-3* mutant (Mut) and wild type control (WT) samples for the seven genes tested. Deletions 1–4 could not be PCR confirmed (see main text) but deletions 5–7 were shown to be false positives as PCR products were amplified from both the control and mutant samples and were of the same size. The PCR oligonucleotides designed to genes within the seven putative deleted regions were: Del 1, *At*1G05135; Del 2, *At*1G15839; Del 3, *At*2G41260; Del 4, *At*3G22142; Del 5, *At*4G33970; Del 6, *At*4G36230; and Del 7, *At*5G07740. (C) Similar to (A), the FN1148 hybridization CGH profile versus control, from Figure 2. Several large potential DNA deletion profiles (~100 kb to 10 mb) were observable on chromosomes 2, 3 and 5. Oligonucleotides were designed to six genes within these deleted regions (Del 1–6) and diagnostic PCR analyses performed. (B) Shows the agarose gel photograph of the PCR products amplified from the FN1148 mutant (Mut) and wild type control (WT) samples for the six genes tested. Deletion 1 was found to be a genuine chromosome 2 deletion of ~4.5 kb (the control PCR product was ~6 kb and the FN1148 mutant product ~1.5 kb), whereas deletions 2–6 were shown to be false positive deletions (the PCR products were amplified from both the control and FN1148 mutant samples and were of the same size). The PCR oligonucleotides designed to genes within the 6 putative deleted regions were: Del 1, *At*2G31080; Del 2, *At*2G33210; Del 3, *At*2G33350; Del 4, *At*3G01345; Del 5, *At*3G57586; and Del 6, *At*5G39770. M is DNA size marker and N is the negative PCR control i.e. without genomic DNA.

**Figure S5**

**Figure S5.** A 108 bp deletion located in the *phyB* gene of the E124 mutant detected with NimbleGen CGH arrays. Scatter plots of the relative DNA hybridisation log_2_ intensities (E124 versus control) of probes representing the *PHYB* gene staggered between 2 to 49 bp. Black dots represent probes and the blue arrow indicates the location of the deletion within the region represented.

**Figure S6**

**Figure S6.** A 28 bp deletion located in the *hy1* gene of the E99 mutant detected with NimbleGen CGH arrays. Scatter plots of the log_2_ relative DNA hybridisation intensities (E99 versus control) of probes representing the *HY1* gene staggered between 2 to 49 bp. Black dots represent probes and the blue arrow indicates the location of the deletion within the region represented.

**Figure S7**

**Figure S7.** A 4 bp deletion located in the *max2* gene of the E207 mutant detected with NimbleGen CGH arrays. Scatter plots of the log_2_ relative DNA hybridisation intensities (E207 versus control) of probes representing the *MAX2* gene staggered between 2 to 49 bp. Black dots represent probes and the blue arrow indicates the location of the deletion within the region represented.

**Figure S8
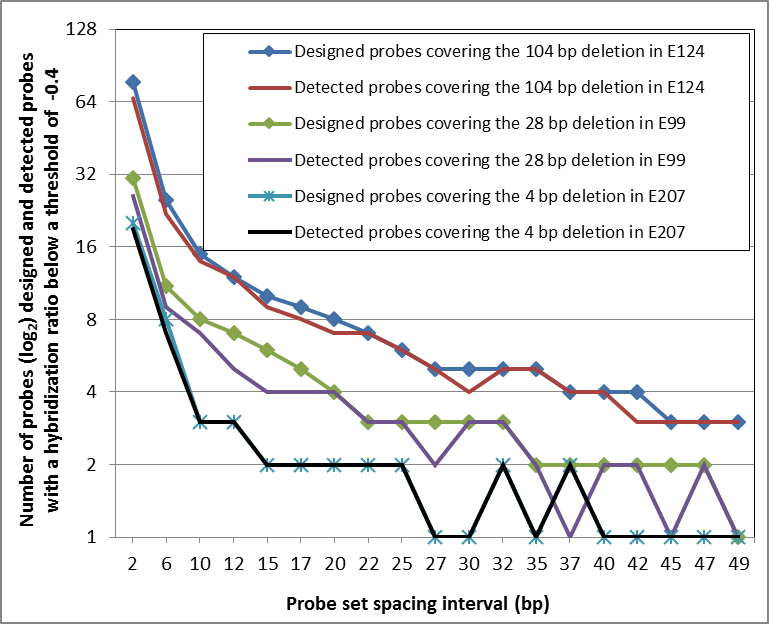
**

**Figure S8.** Design and experimental performance of the NimbleGen CGH array probes. The number of designed and normalised probes with a signal log_2_ ratio of less than -0.4 (y-axis) experimentally detected for genomic deletions present in three *Arabidopsis* mutants (E124, E99 and E207) using probes staggered every 2 to 49 bp (x-axis).

**Table S1**

| **Mutant** | **Mutated gene / accession number** | **Gene coordinates** | **Gene size (bp)** | **Microarray probe design coordinates** | **Deletion mutation coordinates** | **Deletion size (bp)** |
| --- | --- | --- | --- | --- | --- | --- |
| *ga1-3* | *GA1* /*At*4G02780 | Chr4:1,237,881-1,244,766 | 6,885 | Chr4:1,237,381-1,245,266 | Chr4:1,240,624-1,245,674 | 5,051 |
| FN1148 | *HKT1* /*At*4G10310 | Chr4:6,392,001-6,395,663 | 3,662 | Chr4:6,391,501-6,396,163 | Chr4:6,394,514-6,395,036 | 523 |
| E124 | *PHYB* /*At*2G18790 | Chr2:8,147,161-8,151,233 | 4,072 | Chr2:8,146,661-8,151,733 | Chr2:8,149,503-8,149,606 | 104 |
| E99 | *HY1* /*At*2G26670 | Chr2:11,348,877-11,350,472 | 1,578 | Chr2:11,348,377-11,350,972 | Chr2:11,348,880-11,348,907 | 28 |
| E207 | *MAX2* /*At*2G42620 | Chr2:17,763,247-17,765,329 | 2,082 | Chr2:17,762,747-17,765,829 | Chr2:17,763,963-17,763,966 | 4 |

**Table S1.** A table showing the details of known deletions in five *Arabidopsis* mutants used in this study. The size of the fast-neutron induced deletions, the coordinates of the deletions, and the genes affected are listed.

**Table S2**

|  | **Probe staggering and numbers of probes representing each gene** | | | | | | | | | | | | | | | | | | |  |
| --- | --- | --- | --- | --- | --- | --- | --- | --- | --- | --- | --- | --- | --- | --- | --- | --- | --- | --- | --- | --- |
| **Gene (mutant plant line)** | **2** | **6** | **10** | **12** | **15** | **17** | **20** | **22** | **25** | **27** | **30** | **32** | **35** | **37** | **40** | **42** | **45** | **47** | **49** | **Total probes** |
| ***GA1* (*ga1-3*)** |  | 1,051 |  |  |  |  |  |  |  |  |  |  |  |  |  |  |  |  |  | **1,051** |
| ***HKT1* (FN1148)** |  | 280 |  |  |  |  |  |  |  |  |  |  |  |  |  |  |  |  |  | **280** |
| ***PHYB* (E124)** | 2,370 | 817 | 496 | 416 | 333 | 294 | 251 | 228 | 201 | 185 | 168 | 157 | 144 | 137 | 126 | 121 | 113 | 106 | 103 | **6,766** |
| ***HY1* (E99)** | 945 | 371 | 234 | 209 | 168 | 147 | 126 | 115 | 101 | 95 | 85 | 79 | 73 | 69 | 64 | 60 | 57 | 54 | 52 | **3,104** |
| ***MAX2* (E207)** | 1,139 | 450 | 287 | 248 | 199 | 176 | 150 | 136 | 121 | 112 | 101 | 94 | 86 | 82 | 77 | 73 | 67 | 65 | 63 | **3,726** |
|  | **4,454** | **2,969** | **1,017** | **873** | **700** | **617** | **527** | **479** | **423** | **392** | **354** | **330** | **303** | **288** | **267** | **254** | **237** | **225** | **218** | **14,927** |

**Table S2.** The table lists the numbers of custom array probes staggered from 2 bp to 49 bp representing the genes in five *Arabidopsis* deletion mutants used in this study.

**Table S3**

| **Line** | **Location** | **Deletion size (bp)** | **PCR oligonucleotides**  **(5’ to 3’)** | **Sequencing oligonucleotide**  **(5’ to 3’)** | **Sanger sequencing result** |
| --- | --- | --- | --- | --- | --- |
| **E124** | Chr2: 8,149,503 | 104 | TCGATCTCACACTGCGAAAG  AGCAGAAACTCAGCCAGGAG | TCTTTCTCGTGCTTTGAGAGG | Confirmed |
| **E99** | Chr2: 11,348,880 | 28 | Agggcagctcattcaatcac  CACAAGTTGGGAAATTGGAG | tgaatcggctctaaatctcctc | Confirmed |
| **E207** | Chr2: 17,763,963 | 4 | Gctctctctctgcccaacac  CGTCAAGAACCAGCTCCTCT | TACTTCCTCAATGGCCGAGA | Confirmed |

**Table S3.** List of genomic DNA deletion mutations identified and verified in three fast-neutron irradiated mutants. The location (*Arabidopsis* chromosome and TAIR8 genomic co-ordinates) of the mutations are shown. The genomic regions encompassing the deletions in E124, E99 and E207 mutant lines were PCR amplified (Figure S2) and confirmed via Sanger sequencing of PCR products amplified using the listed oligonucleotides.
